# Persistent Organic Contaminants in Dust from the International Space Station

**DOI:** 10.1021/acs.estlett.3c00448

**Published:** 2023-08-08

**Authors:** Stuart Harrad, Mohamed Abou-Elwafa Abdallah, Daniel Drage, Marit Meyer

**Affiliations:** † School of Geography, Earth, and Environmental Sciences, University of Birmingham, Birmingham B15 2TT, United Kingdom; ‡ Queensland Alliance for Environmental Health Sciences (QAEHS), The University of Queensland, 20 Cornwall Street, Woolloongabba QLD 4103, Australia; § Low Gravity Exploration Technology Branch, NASA Glenn Research Center, Cleveland, Ohio 44135, United States

**Keywords:** PBDEs, HBCDD, OPEs, PFAS, PCBs, PAH, dust, spacecraft, ISS

## Abstract

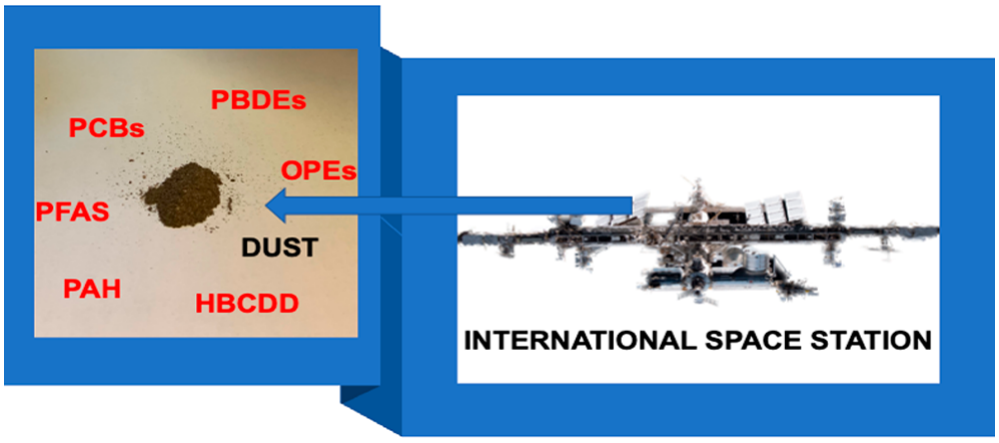

Polybrominated diphenyl ethers (PBDEs), hexabromocyclododecane (HBCDD), “novel” brominated flame retardants (NBFRs), organophosphate esters (OPEs), polycyclic aromatic hydrocarbons (PAH), perfluoroalkyl substances (PFAS), and polychlorinated biphenyls (PCBs) were measured in a composite sample of dust from the International Space Station (ISS). Notwithstanding the unique environment from which the dust originated, while concentrations of all target compound classes frequently exceeded the median values in terrestrial indoor microenvironments in the US and western Europe, ISS dust concentrations were generally within the terrestrial range. The relative abundance of the three HBCDD diastereomers is dominated by γ-HBCDD (96.6% ΣHBCDD). This matches very closely with the commercial mixture added to materials and contrasts with the diastereomer distribution observed in most terrestrial indoor dust samples (in which γ-HBCDD is typically ∼60–70% ΣHBCDD). This suggests conditions inside the ISS do not favor the previously reported photolytically mediated formation in dust of α-HBCDD. Also of note, the concentration of perfluorooctanoic acid (PFOA) in ISS dust (3300 ng/g) exceeds the maximum reported (1960 ng/g) in a 2008 survey of dust from US child daycare centers and homes. This may reflect the widespread use of waterproofing treatments in the ISS to prevent microbial growth. Our findings can inform future material choices for manned spacecraft such as the ISS.

## Introduction

Brominated flame retardants (BFRs) have found substantial use in consumer and commercial applications like electrical and electronic equipment, building insulation, furniture fabrics, and foams. However, an understanding of the potential human health effects has contributed to various jurisdictions banning or limiting severely the manufacture and use of some, exemplified by the listing of hexabromocyclododecane (HBCDD) and the penta-, octa-, and deca-BDE commercial formulations of polybrominated diphenyl ethers (PBDEs) as persistent organic pollutants (POPs) under the UNEP Stockholm Convention.^1^ In addition to PBDEs and HBCDD, other BFRs have been used. Referred to here as “novel” BFRs (NBFRs), examples include hexabromobenzene (HBBz), 1,2-bis(2,4,6-tribromophenoxy) ethane (BTBPE), decabromodiphenyl ethane (DBPDE), 2-ethylhexyl tetrabromobenzoate (EH-TBB), and bis(2-ethylhexyl) tetrabromophthalate (BEH-TEBP). HBBz can be emitted inadvertently from combustion processes, and the others remain in manufacture. To illustrate, EH-TBB and BEH-TEBP are two of the major constituents of the FM-550 commercial product widely used in North America.^2^

Another class of chemicals having wide application as flame retardants (FRs) and plasticizers is organophosphate esters (OPEs). Of these, triphenyl phosphate (TPHP) was an additional major component of FM-550.^2^ The chlorinated OPEs—specifically tris(2-chloroethyl) phosphate (TCEP), tris(1-chloro-2-propyl) phosphate (TCIPP), and tris(1,3-dichloro-2-propyl) phosphate (TDCIPP)—have been extensively applied as FRs in flexible polyurethane foam fillings in domestic and institutional furniture.^2,3^ Moreover, 2-ethylhexyl diphenyl phosphate (EHDPP) and tri-*n*-butyl phosphate (TNBP) have been used as plasticizers and additives to materials like paints and hydraulic fluids.^4,5^ Also listed as POPs are polychlorinated biphenyls (PCBs). In addition to their widespread deployment in applications like permanently elastic sealants, evidence exists of widespread contamination with some PCBs due to their inadvertent formation during production of paint pigments.^7^ Recently, environmental contamination with perfluoroalkyl substances (PFAS) has attracted substantial attention. Their use in consumer goods like fabrics and carpets as stain-repellent formulations^8^ has led to their presence in indoor dust.^8,9^ Moreover, evidence exists of the presence of unintentionally produced chemicals such as polycyclic aromatic hydrocarbons (PAH) in indoor dust. PAHs are emitted as products of incomplete combustion of fossil fuels, as well as being present in fuel.^10^

The International Space Station (ISS) represents a unique indoor environment inhabited by humans for over 20 years since its launch in November 1998. In the context of this study, the vulnerability to fire of spacecraft means very careful attention is paid to the flammability of ISS contents. While many materials used are bespoke, many small, commercial, off-the-shelf components that likely contain FRs and other chemicals are present. Examples include cameras, power tools, mp3 players, tablet computers, medical devices, and clothing.^11^ Moreover, the air inside the ISS is constantly recirculated with ∼8–10 air changes per hour. While CO_2_ and gaseous trace contaminant removal occurs, along with HEPA filtration, the degree to which this removes chemicals such as BFRs on the ISS is unknown. Additionally, high levels of ionizing radiation can cause accelerated aging of materials, including the breakdown of plastic goods into micro- and nanoplastics that can become airborne in the microgravity environment. Hence, we hypothesize that the concentrations and relative abundance of PBDEs, HBCDD, NBFRs, OPEs, PAH, PFAS, and PCBs in ISS dust differ notably from those in dust from terrestrial indoor microenvironments. To our knowledge, this is the first report of the presence of persistent organic chemicals in a nonterrestrial environment.

## Materials and Methods

To test our hypothesis, we measured concentrations of our target chemicals in dust collected from the ISS in 2019 within the Divert Unwanted Space Trash (DUST) experiment. In a microgravity environment, particles do not settle but remain airborne, floating around according to ventilation system flow patterns and eventually depositing on surfaces and air intakes. Consequently, screens covering the ISS HEPA filters accumulate this debris, requiring weekly vacuuming to maintain efficient filtration and reduce the burden on the ventilation fans. The material in ISS vacuum bags comprises previously airborne particles, clothing lint, hair, and other debris generally identified as spacecraft cabin dust. Some vacuum bags were returned to Earth to analyze this unique dust for a wide variety of parameters, with a small aliquot shipped to the University of Birmingham for analysis in the study reported here. On receipt at the University of Birmingham, the sample was sieved through a 125 μm mesh and stored in the dark at −20 °C for ∼4 months until extraction. This yielded a sample mass of just over 200 mg.

### Target Compounds

A full list of compounds targeted in this study is provided as Supporting Information (Table S7).

### Analysis

Full details of methods for extraction, clean up, and analysis of our target compounds are described elsewhere^12−18^ but are also provided as Supporting Information, Tables S1 to S6. In summary, following extraction of 200 mg of dust and extract purification via a combination of SPE (all compounds) and sulfuric acid washes (BFRs and PCBs only), the concentrated extract was analyzed via GC/MS for PBDEs, OPEs, NBFRs, PCBs, and PAHs. Subsequently, the extract was solvent exchanged into methanol and analyzed on a Sciex 5600+ LC-QTOF-MS instrument for PFAS and HBCDDs.

### QA/QC

We analyzed a reagent blank (*n* = 1) consisting of 200 mg of anhydrous sodium sulfate treated as a sample as well as a 200 mg aliquot of SRM2585 (organics in indoor dust). Concentrations detected in the reagent blank were all below detection limits (ranging between 0.1 ng/g to 2.5 ng/g for individual analytes), while those of PBDEs, HBCDD, NBFRs, OPEs, PCBs, PFAS, and PAH fall between 62% and 139% of the certified/indicative values (where available) in SRM2585 (Table S7). We cannot exclude the possibility of sample contamination during shipping from NASA to the lab at Birmingham but consider this unlikely to occur to any substantial degree, as the sample was sealed securely from air contact throughout transit.

## Results and Discussion

Table 1 provides concentrations of all target compounds in the ISS dust sample. Any compounds not listed in Table 1 (five PCBs, three NBFRs, two PFAS, and 10 PAHs) were not detected. Table 1 also provides the median and range of concentrations of the same compounds in illustrative studies of dust from terrestrial indoor microenvironments in the US.

**Table 1:** Concentrations of Target Chemicals (ng g^–1^) in Dust Collected from the ISS^a^ and Comparison with Previous Relevant Studies

compound class	chemical	concentration in ISS dust (ng/g)	median (range) concentration in US house dust (ng/g)
PBDEs	BDE-28	220	4.6 (<1–2300)
	BDE-47	8400	420 (ns-130 000)
	BDE-100	2700	89 (ns-69 000)
	BDE-99	27 000	580 (ns-140 000)
	BDE-154	1900	42 (ns-36 000)
	BDE-153	3600	56 (ns-44 000)
	BDE-183	230	15 (<2–1100)
	BDE-209	18 000	910 (ns-990 000)
NBFRs	HBBz	530	4.7 (<1–1100)
	EH-TBB	790	1200 (<5–130 000)
HBCDDs	α-HBCDD	2300	240 (ns-17 000)
	β-HBCDD	1000	38 (ns-14 000)
	γ-HBCDD	95 000	89 (ns-300 000)
OPEs	EHDPP	59	1100 (ns-42 000)
	TCEP	13	530 (ns-32 000)
	TDCIPP	15	3500 (ns-170 000)
	TCIPP	15	5400 (ns-150 000)
	TPhP	15 700	8100 (ns-110 000)
	TnBP	160	200 (<30–1200)
PCBs	PCB-52	19	6.2 (1.7–28)
	PCB-101	190	8.7 (1.9–29)
	ΣPCBs	210^b^	200 (47–620)^c^
PFAS	PFOS	23	200 (<8.9–12 000)
	PFOA	2,600	140 (<10–1960)
	FOSA	3.7	
	PFHxS	3.7	46 (<13–36 000)
	PFBS	3.5	0.9 (<LOQ-2.6)
	EtFOSE	1.0	<LOQ (<LOQ-94)
	MeFOSE	52	1.0 (<LOQ-9.9)
	PFNA	50	3.9 (1.1–63)
PAH	acenaphthene	930	5.5 (1.3–25)
	phenanthrene	830	95 (25–390)
	anthracene	48	9.6 (2.0–73)
	fluoroanthene	150	76 (12–360)
	pyrene	1600	80 (20–300)

a Only those chemicals present above detection limits in ISS dust listed.

b Sum of PCBs 28, 52, 101, 138, 153, and 180.

c Sum of PCBs 28, 52, 101, 105, 118, 138, 153, and 180.

PBDEs, HBCDDs, NBFRs, and OPEs are from ref (19), *n* = 95, from dust (<150 μm) from floors and elevated surfaces of college dormitories collected in 2015, except for TNBP for Canadian house dust (<500 μm) collected from floors in 2011–2012 from ref (17). PCBs are from ref (12), *n* = 20, from Amarillo and Austin, Texas floor dust (<500 μm) collected in 2006. PFAS is from ref (9) from floor dust (<500 μm) collected in 2013, except for PFOS, PFOA, and PFHxS from ref (8) in vacuum cleaner bag dust (<150 μm) collected in 2000–2001. PAH is from ref (10), *n* = 43, from floor dust samples (<150 μm) collected in 2005–2007 from nonsmoking homes in California.

### PBDEs

Inspection of Table 1 shows that, while concentrations of PBDEs in the ISS dust sample (e.g., BDE-99 = 27 000 ng/g, BDE-209 = 18 000 ng/g) exceed the median reported for US house dust (580 and 910 ng/g for BDE-99 and -209 respectively^19^), they are well within the range reported. That PBDE concentrations in the ISS dust sample fall within the range of concentrations detected in US house dust may reflect use on the ISS of inorganic FRs like ammonium dihydrogen phosphate that are documented as being used on the ISS to flame retard fabrics and webbing.^20^ In addition, guidelines advise that the use of any commercial off-the-shelf items such as electronics should not exceed 1 h/day, and must be stowed away in nonflammable stowage containers/compartments after each use.^11^ The congener pattern dominated by BDEs 99, 209, and 47 with moderate contributions from BDEs 100, 153, and 154 suggests the sources of PBDEs in the ISS dust are articles treated with one (or both) of the Penta-BDE and Deca-BDE commercial products.^21^ For Penta-BDE this is likely flexible polyurethane foam used in furniture and mattresses, while the Deca-BDE product was used as a back-coating for furniture/mattress fabrics as well as in hard plastic housings for electrical and electronic goods. The use of foam is critical in packing experiment payloads and delicate equipment to survive the difficult launch environment; therefore, there are large amounts in every cargo resupply vehicle. This foam is stored until it can be trashed, making it a ubiquitous material on the ISS. Commercial off-the-shelf electrical goods with plastic housings are present, but there are some limitations. In general, smaller items can be used as-is, but some larger items require repackaging or covering the plastic with metallized tape to reduce the flammable surface area. Nonetheless, there is no limit on the quantity of such items, which have been present in the cabin for decades. In contrast, the lower abundance of BDE-183 suggests articles containing the Octa-BDE formulation are not abundant on the ISS, as this congener is generally recognized as the dominant congener present in the Octa-BDE product.^21^

### HBCDD

The concentration of ΣHBCDD in ISS dust (98 000 ng/g) exceeds substantially the median concentration reported in US house dust.^19^ However, it is lower than the highest value detected in US house dust (300 000 ng/g), and similarly elevated concentrations have also been reported in several samples of house dust from the UK, from 100 000 ng/g up to 570 000 ng/g.^22,23^ As well as the elevated concentration of ΣHBCDD, the relative contribution of the three HBCDD diastereomers in the ISS dust (96.6% γ-, 2.3% α-, and 1.0% β-HBCDD) is noteworthy. Studies of indoor dust from homes in Canada, the UK, and the US^19,22,23^ report in most samples a greater relative abundance of the α- and β- diastereomers than observed in the ISS dust. Moreover, even in the most contaminated dust sample reported (in a UK home), the relative abundance is α- (23%), β- (11%), and γ- (66%).^23^ The predominance of γ-HBCDD observed in ISS dust is consistent with that reported in the HBCDD commercial product.^24^ Studies suggest the shift from predominantly γ-HBCDD toward more α- and to a lesser extent β-HBCDD can occur via photolytic isomerization following emission to dust.^25^ The absence of such a shift in ISS dust suggests that the unique environment of the ISS limits such photolytically mediated isomerization.

### NBFRs

Of the five NBFRs targeted, three were not detected (BEH-TEBP, BTBPE, and DBDPE). Concentrations of EH-TBB and HBBz were 790 and 530 ng/g, respectively. Compared to concentrations reported for US house dust, those of EH-TBB and HBBz are respectively below and above the median reported previously, but both fall comfortably within the range reported.^19^ Contrastingly, the absence of BEH-TEBP, BTBPE, and DBDPE in ISS dust differs substantially from the detection of all three of these NBFRs in US house dust at median concentrations of 1200 ng/g, 9.4 ng/g, and 100 ng/g, respectively.^19^ Overall, the relatively low concentrations of our target NBFRs in the ISS dust compared to the more recent terrestrial data likely reflect the age of the putative sources present on the ISS, such that they are less likely to have been treated with such alternatives to PBDEs and HBCDD, both unrestricted at the time of ISS construction in the late 1990s. The presence of EH-TBB and TPHP—see below—is indicative of the proprietary product FM-550 reported to be used to meet fire retardancy regulations in items like couches.^2^ However, we did not detect BEH-TEBP which has also been reported as a main component of FM-550. We cannot provide a definitive explanation for this observation.

### OPEs

The predominant OPE in ISS dust is TPHP (16 000 ng/g). This exceeds the median reported for US house dust but is comfortably within the range reported for such samples.^19^ As noted above, the presence of TPHP in ISS dust may arise from its use in the FM-550 flame retardant formulation used in furniture foam in the US. Alternatively, TPHP has been reported to be used as a plasticizer in PVC electrical cables,^26^ present in commercial off-the-shelf items on ISS. In contrast, concentrations of all other target OPEs in ISS dust are much lower (≤160 ng/g). TNBP is present in ISS dust at a concentration very similar to the median for Canadian house dust.^17^ Plausible potential sources of TNBP in the ISS stem from its use as a plasticizer and in hydraulic fluids.^4^ Interestingly, concentrations of the three chlorinated OPEs (TCEP, TCIPP, and TDCIPP) used widely as FRs in flexible and rigid polyurethane foam found in seating cushions and building insulation, respectively,^2,3^ are all much lower than in studies of indoor dust from US homes. The low concentrations of these chlorinated OPEs, alongside the higher concentrations of PBDEs associated with the penta-BDE product, are consistent with likely ongoing use of the penta-BDE product during the mid-to-late 1990s during ISS construction.

### PCBs

Of the seven PCBs targeted, we detected only PCB 52 (19 ng/g) and PCB 101 (190 ng/g). The concentration of PCB 52 was at the high end of the range for US house dust,^8^ while that of PCB 101 was ∼7 times higher than the maximum reported.^12^ However, the absence of the other five target PCBs means ΣPCB concentrations in ISS dust (210 ng/g) only just exceed the median of 200 ng/g reported for US house dust. That the PCB concentrations in the ISS dust are not especially elevated is illustrated by the higher concentrations detected in two studies of house dust from two areas suspected impacted by PCB point sources in Massachusetts, US,^27,28^ in which maximum concentrations of 3600 ng/g (ΣPCB) and 16 000 ng/g (PCB 52) were detected. Unfortunately, both of these studies provided very limited information on concentrations of individual PCBs, so a more detailed comparison is difficult. The congener pattern detected in ISS dust in which PCB 101 (a pentachlorobiphenyl) predominates followed by PCB 52 (a tetrachlorobiphenyl) is unusual and does not resemble those anticipated in indoor dust as a result of emissions from any of the main commercial PCB products used in the US—i.e., Aroclors—or indeed the Sovol formulation originating from the erstwhile Soviet Union.^29^ However, a possible source of PCBs consistent with this unusual pattern and the detection of HBBz (see NBFRs discussion) is their inadvertent formation during the production of azo and phthalocyanine paint pigments.^7^

### PFAS

The predominant PFAS in ISS dust is perfluorooctanoic acid (PFOA) at 3300 ng/g. The next most abundant compound is MeFOSE at 52 ng/g, closely followed by PFNA at 50 ng/g. Compared to their concentrations in US house dust,^8,9^ the concentrations of most target PFAS are within the same range. However, it is noticeable that PFOA is present in ISS dust at a concentration slightly exceeding the maximum reported (1960 ng/g) in a survey of dust from US child daycare centers and homes.^8^ The elevated PFOA concentration in ISS dust may reflect the application of the 3M waterproofing formulations Scotchguard 4101 and 4106-PF at various points during the lifetime of the ISS to, e.g., prevent microbial growth.^20^ Another potential source of PFAS in the ISS is Braycote 601 EF vacuum grease, also used on exercise machines, as a corrosion inhibitor, and in larger quantities to maintain the hatch seals of the US on-orbit segment (USOS) of the ISS. The manufacturer of this product specifies that it is a perfluorinated polyether.^30^ Also of note, while commercial items with flammable surfaces must ideally be covered with a nonflammable tape, a cited alternative to such tape is a nonflammable barrier material such as a fluoroelastomer.^11^

### PAH

Of the 15 PAH targeted, only five were detected in order of decreasing concentration: pyrene (1600 ng/g), acenaphthene (930 ng/g), phenanthrene (830 ng/g), fluoranthene (150 ng/g), and anthracene (48 ng/g). All other target PAHs were <40 ng/g. Concentrations in the ISS dust all exceed the median concentrations in dust from nonsmoking homes in the US.^10^ Moreover, while those of anthracene and fluoranthene are within the range reported for US house dust, those of the other three detected PAHs all exceed the maximum found in house dust.

### Sources of Contamination

The sources of contamination on the ISS are difficult to trace. With respect to the origin of the dust present in the ISS, we note the observation in an earlier paper by one of the authors that “the bulk of the aerosol emissions on ISS are carbonaceous particles, and human skin flakes are the largest proportion”.^31^

Materials of common items are generally known; however, there are many unique payload experiments with exotic materials. Occasionally the vacuum cleaner is used to clean up materials after an experiment is performed, resulting in higher concentrations of some materials not indicative of typical airborne material concentrations. Quantities of these materials are not tracked, and the sampling method via vacuuming was not quantitatively controlled in terms of duration of dust buildup or by ISS location. One possible source of elevated concentrations of BFRs in the dust sample stems from the practice of vacuuming wall panels and acoustic insulation, which results in larger relative quantities that cannot be correlated with terrestrial human exposure. Consequently, our analysis of these data cannot yield direct insight into the origins of dust contamination with any of the target compounds in the ISS dust. The results do have implications for future space stations and habitats, where it may be possible to exclude many contaminant sources by careful material choices in the early stages of design and construction.

## Supporting Information Available

Details of analytical methods, QA/QC data, and additional references (Tables S1–S7) (PDF)
