# Supplementary material for: Persistent Organic Contaminants in Dust from the International Space Station
Source: Environ Sci Technol Lett. 2023 Aug 8;10(9):768–72. doi: 10.1021/acs.estlett.3c00448 (PMC10501190; doi:10.1021/acs.estlett.3c00448)
Supplement: Supplementary file 1 — ez3c00448_si_001.pdf [file ez3c00448_si_001.pdf]

Supporting Information for

PERSISTENT ORGANIC CONTAMINANTS IN DUST FROM THE INTERNATIONAL SPACE STATION

Stuart Harrad, Mohamed Abou-Elwafa Abdallah, Daniel Drage, Marit Mayer

7 pages, 1 method description, 7 tables

Extraction

200 mg of dust was accurately weighed into a clean glass tube. The dust was spiked with 50 µL of an internal standard solution containing 1 ng/µL of d_{10}-acenapthene, d_{10}-fluorene, d_{10}-phenanthrene, d_{10}-anthracene, d_{10}-fluoroanthene, d_{10}-pyrene, d_{12}-benzo-(a)-anthracene, d_{12}-chrysene, d_{12}-benzo-(b)-fluoranthene, d_{12}-benzo-(k)-fluoranthene, d_{12}-benzo-(a)-pyrene, d_{12}-indeno-(1,2,3-cd)-pyrene, d_{12}-benzo-(g,h,i)-perylene, PCB-34, PCB-62, PCB-119, PCB-131, PCB-173, BDE-77, BDE-128, $^{13}C_{12}$-α-HBCDD, $^{13}C_{12}$-β-HBCDD, $^{13}C_{12}$-γ-HBCDD, d15-tri-phenyl-phosphate, d_{15}-TDCIPP, and d_{12}-TCEP; and 2 ng/µL of $^{13}C_{12}$-BDE-209.

5 mL of hexane:acetone (1:1, v/v ratio) was added to the glass tube which was vortexed for 2 minutes prior to ultrasonication for 30 mins at 25 °C. The extract was centrifuged for 5 minutes at 2000 G and the supernatant was transferred to a clean glass tube. The extraction process was repeated a further two times (three times in total) and all three supernatants combined. 15 mL extracts were concentrated to incipient dryness at 38 °C under a gentle stream of nitrogen, and immediately reconstituted in 1 mL hexane. The extract was divided into two fractions 50/50 volumetrically. The first extract (Extract A) was cleaned-up in preparation for determination of PCBs, PAHs, PBDEs, HBCDDs, EH-TBB, HBBz and OPFRs. The second Extract (Extract B) was cleaned up for PFAS.

Clean-up

Extract A was transferred onto a florisil SPE cartridge (Hypersep, 1g/6mL, Thermo Fisher Scientific), which was preconditioned with 10 mL Acetone and 10 mL hexane.

The extract was fractionated into two fractions. The first (F1) a was eluted with 8 mL hexane and retained. The second fraction (F2) was eluted with 10 mL acetone. 100 µL of nonane was added to F2 as a keeper solvent and it was concentrated to 100 µL at 38 °C under a gentle stream of nitrogen and transferred to an autosampler vial and determination of OPFRs. After determination of OPFRs, F1 was concentrated to 100 µL at 38 °C under a gentle stream of nitrogen, and was recombined with F2 into the autosampler vial and was injected onto GC/MS for the determination of PAHs.

After PAH analysis, samples were transferred into clean glass tubes and diluted to 2 mL in hexane. Approximately 2 mL >95% sulfuric acid was added and the tube was vortexed for 2 mins and centrifuged for 3 minutes at 2000 G. The supernatant hexane layer was transferred to a clean tube along with 2 x 1 mL rinses. The clean extract was concentrated to approximately 50 µL at 40 °C under a gentle stream of nitrogen and transferred to GC vials ready for analysis of PCBs, PBDEs, HBBz and EH-TBB via GC/MS; and HBCDD via LC-TOF/MS.

For the determination of PFAS, Extract B was solvent exchanged into approximately 0.5 mL methanol. It was loaded onto an ENVI-Carb SPE cartridge (3 mL, 500 mg, Sigma Aldrich), pre-conditioned with 6 mL of methanol (0.1% NH4 OH) and 3 mL of methanol. Target PFASs were eluted with 3 mL of methanol (0.1% NH4OH). Eluates were concentrated to approximately 0.5 mL, passed through a 0.2 µm syringe filter, concentrated to 100 µL, and transferred to autosampler vials ready for analysis.

Chemical Analysis

PAHs

Samples were analysed for PAHs on an Agilent 6850 GC coupled to an Agilent 5975C mass spectrometer (MS). The transfer line and source were set to 230 °C. The MS was operated in EI using

SIM. 1 µL of extract was injected in splitless mode at 300 °C. Helium was used as a carrier gas at a flow rate of 1 mL/min. Separation was achieved on a Restek Rxi-5Sil-ms (30 m x 0.25 mm x 0.25 µm film thickness) column. The initial GC oven temperature was 70 °C for 1 min. It was then ramped at 6 °C/min to 120 °C followed by 4 °C/min to 300 °C and held for 5 minutes.

Table S1 details monitoring ions for PAH analysis:

Native PAH	Monitoring ion (m/z)	Deuterated PAH	Monitoring ion (m/z)
**Acenaphthylene**	152	**d_{10}-Acenaphthene**	164
**Acenaphthene**	153		
**Fluorene**	166	**d_{10}-fluorene**	176
**Phenanthrene**	178	**d_{10}-phenanthrene**	188
**Anthracene**	178	**d_{10}-anthracene**	188
**Fluoranthene**	202	**d_{10}-fluoranthene**	212
**Pyrene**	202	**d_{10}-pyrene**	212
**Benzo-(a)-anthracene**	228	**d_{12}-benzo-(a)-anthracene**	240
**Chrysene**	228	**d_{12}-chrysene**	240
**Benzo-(b)-fluoranthene**	252	**d_{12}-benzo-(b)-fluoranthene**	264
**Benzo-(k)-fluoranthene**	252	**d_{12}-benzo-(k)-fluoranthene**	264
**Benzo-(a)-pyrene**	252	**d_{12}-benzo-(a)-pyrene**	264
**Indeno-(1,2,3-cd)-pyrene**	276	**d_{12}-indeno-(1,2,3-cd)-pyrene**	288
**Dibenzo-(a,h)-anthracene**	278	**d_{12}-benzo-(g,h,i)-perylene**	288
**Benzo-(g,h,i)-perylene**	276		

OPFRs

Final sample extracts were analysed via GC-EIMS using an Agilent 5975C MS fitted with a DB-5ms column (30 m, 0.25 mm id, 0.25 µm film thickness). The GC temperature programme was 90 °C, hold for 1.25 min, ramp 10 °C/min to 170 °C, ramp 5 °C/min to 240 °C, hold for 10 min, ramp 20 °C/min to 310 °C, hold for 10 min. The mass spectrometer was operated in selected ion electron ionisation mode, with Table S2 listing the ions monitored for each targeted compound.

Table S2 – Details of the monitoring ions used for OPFR determination

**Compound**	**Primary ion (m/z)**	**Secondary ion (m/z)**
**TCEP**	249	251
**TCIPP**	277	279
**TPHP**	326	325
**TDCIPP**	381	379
**EHDPP**	251	250
**D_{15}-TDCIPP**	486	484
**D_{12}-TCEP**	261	263
**D_{15}-TPHP**	341	339

PCBs

Samples were analysed for PCBs on an Agilent 6850 GC coupled to an Agilent 5975C MS. The MS was operated in EI using SIM. Both the source and transfer line were set to 280 °C. 1 µL of

extract was injected in splitless mode at 250 °C. Helium was used as a carrier gas at a flow rate of 1 mL/min. Separation was achieved on a Restek Rxi-5Sil-ms (30 m x 0.25 mm x 0.25 µm film thickness) column. The GC oven was initially set to 80 °C and held for 2 mins. It was then ramped at 20 °C/min to 180 °C and held for 0.5 min followed by a ramp to 290 °C at 10 °C/min and held for 8 minutes.

Table S3 monitoring ions used for PCB determination:

PCB	Primary ion (m/z)	Secondary ion (m/z)
**28**<br>**34 (IS)**	255.96	257.96
**52**<br>**62 (IS)**	291.92	289.92
**101**<br>**118**<br>**119 (IS)**	325.88	327.88
**138**<br>**131 (IS)**<br>**153**	359.84	361.84
**173 (IS)**<br>**180**	393.80	395.80

PBDEs and NBFRs

PBDEs and NBFRs were determined on a Thermo TRACE 1310 GC coupled to a Thermo ISQ MS. The instrument was operated in EI mode using SIM. 1 µL of the purified extract was injected for analysis using a programmable temperature vaporiser (PTV) onto a Thermo Trace Gold TG-5ms (15 m x 0.25 mm x 0.25 µm film thickness) column. Helium was used as the carrier gas at a flow rate of 1.5 mL/min. The GC oven was initially set to 100 °C and held for 0.5 min. It was then ramped to 250 °C at 20 °C/min, then 5 °C/min to 270 and then 20 °C to 305 °C and held for 15 minutes.

Table S4 monitoring ions for PBDEs & NBFRs

PBDE	Primary ion (m/z)	Secondary ion (m/z)
**BDE-28**	405.8	407.8
**BDE-47**<br>**BDE-77 (IS)**	485.7	483.7
**BDE-100**<br>**BDE-99**	403.8	405.8
**BDE-154**<br>**BDE-153**<br>**BDE-128 (IS)**	483.7	485.7
**BDE-183**	561.5	563.5
**BDE-209**	799.4	801.4
**$^{13}C_{12}$-BDE-209**	809.4	811.4
**EH-TBB**	420.7	418.7
**HBBz**	551.5	549.5
**BTBPE**	356.8	358.8
**BEH-TEBP**	464.7	466.7
**DBDPE**	484.6	486.6

HBCDDs

HBCDDs (α-, β-, and γ-HBCDD) were measured using a Sciex Exion UPLC coupled to a Sciex 5600+ Triple TOF MS as outlined in Drage et al. (2020). Determination of HBCDD isomers (α-, β- and γ-). Chromatographic separation was achieved with a AccucoreTM RP-MS column (100 × 2.1 cm, 2.6 µm, Thermo Scientific, Bremen, Germany) using a mobile phase of high purity water (Optima grade, Fisher Scientific (mobile phase A)) and methanol (Optima grade, Fisher Scientific (mobile phase B)). The LC program commenced with 25% B and was ramped to 50% over 1 min, and increased linearly to 100% B over 5 min and held for a further 1 min. The mobile phase composition was returned to 25% B and held for 1 min to equilibrate for the next sample. The overall method duration was 8 min with a flow rate of 0.3 mL/min. The injection volume was 5 µL and the column oven was maintained at 35 °C throughout. Target analytes were identified using a combination of correct retention time and two accurate m/z values. A mass error tolerance of 25 ppm was applied. Native HBCDDs were quantified with m/z 640.6370 and confirmed with 642.6350. $^{13}C_{12}$-HBCDDs were quantified with m/z 652.6773 and confirmed with 654.6753.

PFAS

PFAS (PFOA, PFOS, PFNA, PFHxS, PFBS, FOSA, EtFOSA, MeFOSA, EtFOSE, MeFOSE) were determined in accordance with the procedures described in Harrad et al (2019a; 2019b) on a Sciex Exion HPLC coupled to a Sciex 5600+ triple TOF MS. Ten microliters of extract was injected onto a Raptor C18 column (1.8 µm particle size, 50 mm length, 2.1 mm internal diameter, Restek). Details of the HPLC program are provided in Table S5.

Table S5 HPLC conditions for determination of PFAS

**Mobile Phase**	A: 5 mM ammonium formate in water<br>B: 5 mM ammonium formate in methanol		
**Column**	Restek Raptor C18 column 1.8 µm particle size, 50 mm length, 2.1 mm internal diameter		
**Flow Rate**	0.4 mL/min		
**Gradient**	**Time**	**A (%)**	**B (%)**
	0.00	80	20
	6	5	95
	6.5	5	95
	6.51	80	20
	8	80	20

The TOFMS was equipped with a Turbo V source which was operated in negative mode using electrospray ionisation at a voltage of -4,500 V. The curtain gas was set at 25 psi, whilst the nebulizer gas (source gas 1) was set at 25 psi and the drying gas (source gas 2) at 35 psi. The CAD gas was set to medium and temperature was 450 °C. The MS data was acquired using automatic information dependent acquisition (IDA) with two experiment types: (i) survey scan, which provided TOF-MS data; and (ii) dependent product ion scan using a collision energy of -40V and a collision a spread of 30 V. Quantification of individual PFAS was performed in Multiquant 2.0 using MS/MS transitions and retention time (Table S6) for identification.

Table S6 – MS/MS transitions for determination of PFAS

**Compound**	**MS/MS Transition**	**Retention Time**

**Native Compounds**		
PFOA	413.16 → 369.12	4.51
PFOS	499.17 → 79.98	5.09
PFNA	463.16 → 419.19	4.89
PFHxS	399.13 → 79.98	3.89
PFBS	299.10 → 79.98	2.85
FOSA	498.17 → 77.97	5.04
EtFOSA	526.23 → 169.06	5.91
MeFOSA	512.17 → 169.07	5.97
EtFOSE	616.27 → 59.11	6.01
MeFOSE	630.33 → 59.11	6.05
**Internal Standards**		
$^{13}C_8$PFOA	421.3 → 377.12	4.50
$^{13}C_8$-PFOS	507.1 → 79.98	5.08
$^{13}C_5$-PFNA	468.1 → 419.19	4.89
$^{18}O_2$-PFHxS	403.4 → 79.98	3.89
d_3-MeFOSA	515.0 → 169.1	5.92
d_7-MeFOSE	609.0 → 59.1	6.01

Table S7 Measured and certified concentrations of target analytes in SRM-2585

	Measured	Certified/Indicative	Measured value as % of certified/indicative value
BDE-28	38.4	46.9	82
BDE-47	535	497	108
BDE-100	131	145	90
BDE-99	824	892	92
BDE-154	87.7	83.5	105
BDE-153	165	119	139
BDE-183	37.7	43	88
BDE-209	2502	2510	100
α-HBCDD	26.4	21.2	
β-HBCDD	4.64	4.3[a]	108
γ-HBCDD	135	120[a]	
HBBz	3.4	2.8[b]	121
EH-TBB	36.8	38.8[b]	95
PFOA	620	579[c]	107
FOSA	6.1	9.4[c]	
PFHxS	1660	1400[c]	
PFOS	2360	2214[c]	
PFBS	81	65[c]	
EtFOSA	0.38	n.r	
MeFOSA	261	n.r	
EtFOSE	134	n.r	
PFNA	59.6	96.5[c]	62
MeFOSE	1380	n.r	
TCEP	681	925	74

TDCIPP	1140	1900[d]	60
TCIPP	849	1220	70
TPhP	1140	1190	96
EHDPP	890	n.a	
PCB 28	12.0	13.4	90
PCB 52	19.8	21.8	91
PCB 101	28.7	29.8	96
PCB 138	24.1	27.6	87
PCB 153	38.9	40.2	97
PCB 180	16.9	18.4	92

[a]Aballah et al 2008
[b]Fan et al. 2016
[c]Reiner et al. 2015
[d]Abdallah & Covaci 2014

References for SI

- Drage, D.S., Waiyarat, S., Harrad, S., Abdallah, M.A.-E., Boontanon, S.K. Temporal trends in concentrations of legacy and novel brominated flame retardants in house dust from Birmingham in the United Kingdom, *Emerging Contaminants*., **2020**, 6, 323-329
- Harrad, S.; Drage, D. S.; Sharkey, M.; Berresheim, H. Brominated flame retardants and perfluoroalkyl substances in landfill leachate from Ireland. *Sci. Total Environ.*, **2019a**, 695, 133810.
- Harrad, S.; Wemken, N.; Drage, D. S.; Abdallah, M.A.-E.; Coggins, A. M. Perfluoroalkyl substances in drinking water, indoor air and dust from Ireland: implications for human exposure. *Environ. Sci. Technol.*, **2019b**, 53, 13449-13457.
- Abdallah, M. A-E.;. Harrad, S.; Ibarra, C.; Diamond, M.; Melymuk, L., Robson, M.; Covaci, A. Hexabromocyclodocanes in indoor dust from Canada, United Kingdom and United States. *Environ. Sci. Technol.,* **2008**, *42*, 459-464.
- Fan, X.; Kubwabo, C.; Rasmussen, P.E.; Wu, F. *Environ Sci Pollut Res* **2016**, *23*, 7998–8007.
- Reiner, J. L.; Blaine, A. C.; Higgins, C. P.; Huset, C.; Jenkins, T. M.; Kwadijk, C. J. A. F.; Lange, C. C.; Muir, D. C. G.; Reagen, W. K.; Rich, C.; Small, J. M.; Strynar, M. J.; Washington, J. W.; Yoo, H.; Keller, J. M. Polyfluorinated substances in abiotic standard reference materials. *Anal. Bioanal. Chem.,* **2015**, *407*, 2975-2983.
- Abdallah, M. A.; Covaci, A. Organophosphate Flame Retardants in Indoor Dust from Egypt: Implications for Human Exposure. *Environ Sci Technol.,* **2014**, *48*, 4782−4789.
